# Vitamin D status and its determinants among Chinese infants and toddlers in Hong Kong

**DOI:** 10.1007/s00394-025-03691-0

**Published:** 2025-06-11

**Authors:** Keith T. S. Tung, Hung Kwan So, Chen Chen, Joanna Y. L. Tung, Hing Wai Tsang, Rosa S. Wong, Sophie S. F. Leung, Calvin K. M. Cheung, Albert Lee, Jason C. S. Yam, Wing Cheong Leung, Patrick Ip

**Affiliations:** 1 Department of Paediatrics and Adolescent Medicine, School of Clinical Medicine, The University of Hong Kong, Hong Kong SAR, China; 2 Department of Paediatrics, Hong Kong Children’s Hospital, Hospital Authority, Hong Kong SAR, China; 3 Department of Special Education & Counselling, The Education University of Hong Kong, Hong Kong SAR, China; 4 Department of Paediatrics, Faculty of Medicine, The Chinese University of Hong Kong, Hong Kong SAR, China; 5 Jockey Club School of Public Health and Primary Care, The Chinese University of Hong Kong, Hong Kong SAR, China; 6 Department of Ophthalmology and Visual Sciences, The Chinese University of Hong Kong, Hong Kong SAR, China; 7 Department of Obstetrics and Gynaecology, Kwong Wah Hospital, Hong Kong SAR, China

**Keywords:** Infants, Toddlers, 25(OH)D, Vitamin D deficiency, Chinese

## Abstract

**Purpose:**

Vitamin D is a micronutrient that is necessary for bone health as well as the regulation of mineral homeostasis. This study aims to assess the vitamin D status and its determinants among infants and toddlers in Hong Kong, two of the most vulnerable groups at risk of vitamin D insufficiency.

**Methods:**

A multi-centre cross-sectional study recruiting 887 infants and toddlers was conducted. A comprehensive assessment on potential factors that can influence vitamin D status as well as serum vitamin D concentration was conducted for all participants. Regression analyses were conducted to assess the factors contributing to the vitamin D insufficiency risk.

**Results:**

124 (14.0%) infants and toddlers were vitamin D insufficient and 44 (5.0%) were vitamin D deficient, with higher prevalence observed among younger infants (aged 2 to 6 months). Majority did not consume any vitamin D-containing supplement (86.5%) which was associated with higher risk of vitamin D insufficiency (aOR = 2.16, *p* = 0.017). An increment of 100IU in total daily vitamin D intake lowered the risk of vitamin D insufficiency (aOR = 0.50, *p* < 0.001) in older infants and toddlers. Being breastfed increased vitamin D insufficiency risk in younger infants (aOR = 24.91, *p* < 0.001) and in older infants and toddlers (aOR = 7.36, *p* < 0.001). Further analyses identified distinctive pattern among factors that can influence 25(OH)D3, 25(OH)D2, and 3-epi-25(OH)D3 concentrations.

**Conclusion:**

Low total vitamin D intake and lack of supplementation practice were the key contributing factors to vitamin D insufficiency among infants and toddlers in Hong Kong. Detailed guidelines and support should be provided to meet their respective daily vitamin D intake requirement to further tackle the vitamin D insufficiency and deficiency among these vulnerable populations.

## Introduction

Vitamin D, a fat-soluble steroid hormone, plays a vital role in maintaining calcium and phosphorus homeostasis during the whole lifespan, especially in childhood [1]. In addition, previous studies have suggested that vitamin D deficiency is also associated with increased risks of developing other extra-skeletal physical and mental health problems in children [2–4]. In particular, infants and toddlers are vulnerable age groups of vitamin D deficiency. Despite their adequate needs for vitamin D for their rapid growth, limited sunlight exposure, restricted dietary options and inadequate vitamin D storage make them more vulnerable to vitamin D deficiency [5]. Chronic vitamin D deficiency during early childhood may cause rickets, resulting in growth retardation, skeletal deformities and adverse brain development in children, and even increased risks of hip fracture later in life, and adverse effects on the immune system and psychiatric conditions [6–8]. Therefore, it is crucial to determine how different risk factors influence the vitamin D status in infants and toddlers.

Vitamin D can be generated in the skin upon exposure to ultraviolet light or ingested from dietary sources and supplements [9]. Therefore, factors associated with inadequate sun exposure and dietary limitations are primary contributors to vitamin D deficiency. Vitamin D status in infants and toddlers is affected by geographical conditions, socio-cultural factors, and genetic inheritance [10, 11]. Although this has been extensively studied in various countries and cities, there is a lack of data among Chinese infants and toddlers in Hong Kong, making it crucial to investigate local vitamin D status.

Furthermore, there is a lack of evidence regarding how different vitamin D metabolites contribute to the total vitamin D status in early childhood, particularly in terms of the risk of vitamin D deficiency [12–14]. Most previous studies on vitamin D status used a total 25(OH)D concentration as a golden indicator to represent the total bioactive forms of vitamin D. Total 25(OH)D is mainly determined by two main forms: 25(OH)D3 and 25(OH)D2, but the interpretation of serum 25(OH)D in infants is complicated by the presence of a C3-epimeric form of 25(OH)D3 (3-epi-25(OH)D3), which contributes up to 61% of total 25(OH)D in infants up to first year of life [15]. However, this vitamin D metabolite is less potent compared to 25(OH)D3 and often be erroneously included as a total 25(OH)D [15, 16].There is also an increasing need to disentangle 25(OH)D2 and 25(OH)D3 to better understand the role of vitamin D2 and vitamin D3 on total vitamin D status. 25(OH)D3 is derived from cutaneous synthesis, animal source food and supplements, while 25(OH)D2 is synthesized from vitamin D2 from plant sources and supplements [17]. Although vitamin D3 supplements are often preferred over vitamin D2, the utilization of vitamin D2 products and foods containing vitamin D2 (such as mushrooms and yeast) is common in China [18]. A study based on Chinese National Nutrition and Health Surveillance found that children aged 3–5 years without 25(OH)D2 were 4.2 times more likely to have vitamin D deficiency compared to those with 25(OH)D2. This suggested that vitamin D2 may play a role in preventing vitamin D deficiency in Chinese children [19]. A reciprocal regulatory mechanism between 25(OH)D2 and 25(OH)D3 concentrations was also found that changes in 25(OH)D2 and 25(OH)D3 concentrations were correlated with each other [20]. Therefore the proportions of 25(OH)D2 and 25(OH)D3 may impact the effect of total 25(OH)D and the underlying biological explanation for this observation is yet to be clarified [21]. In addition, 25(OH)D2 and 25(OH)D3 might have different associations with various diseases including cardiovascular disease and diabetes in children and adults [22, 23]. Therefore, simultaneously quantifying 25(OH)D3, 25(OH)D2, and 3-epi-25(OH)D3, as well as examining their associated factors, is crucial for accurately classifying vitamin D deficiency and gaining a more comprehensive understanding of their roles, interactions, and effectiveness in relation to various health outcomes in young children.

Our study has 3 objectives (1) to report the prevalence of vitamin D deficiency and insufficiency in infants and toddlers in Hong Kong; (2) to determine the risk factors associated with vitamin D deficiency and insufficiency; (3) to investigate whether the concentrations of 25(OH)D2, 25(OH)D3 and 3-epi25(OH)D3 are influenced by the identified vitamin D risk factors.

## Method

### Study design and participants

This was a multi-centre cross-sectional observational study conducted among Chinese infants (2 to 11 months) and toddlers (12 to 23 months). All study participants were recruited from 6 Maternal and Child Health Centres in different districts in Hong Kong by stratified sampling in the period between July 2019 and November 2021. Infants and toddlers with any major congenital malformations, or with any chronic medical problems, or those born premature or with birth weight of less than 2500gm, were excluded from this study to minimise the confounding effects of health conditions. Eligible mother-child dyads were approached and recruited at the Maternal and Child Health Centres, where more than 90% of local children attend for vaccination and child health services. Upon obtaining informed consent, the mothers were asked to complete a questionnaire on demographics, dietary record, supplementation practice, mode of feeding and sunscreen usage of their child. Non-fasting peripheral blood samples were collected via venepuncture from the infants and toddlers for the assessment of vitamin D status. All samples were processed on the same day by trained staff to extract the serum for vitamin D status assessment. An incentive of HKD200 (approximately USD 25.6) supermarket voucher was given to the participants to cover cost of transportation upon completion of the study assessment. To ensure an even age distribution across 2 to 23 months, stratified recruitment by age group was adopted.

## Measures

### Assessment of vitamin D status

Total serum 25-hydroxyvitamin D [25(OH)D] concentration, defined as the sum of 25(OH)D3 and 25(OH)D2 minus 3-Epi-25(OH)D3 concentrations, was used to evaluate the vitamin D status of the child participants. Participants with total serum 25(OH)D concentration less than 25 nmol/L were considered as “vitamin D deficient” and those with total serum 25(OH)D concentration less than 50 nmol/L but higher or equal to 25 nmol/L were considered as “vitamin D insufficient”. These cut-offs used have been recommended in the Consensus Vitamin D position statement from the UK National Health Service [24].

Serum was first separated from the collected blood samples to determine the total 25(OH)D concentration by liquid chromatography-tandem mass spectrometry method (LC-MS/MS). The AB Sciex Triple Quad QTRAP 5500 + LC-MS/MS system (AB Sciex Pte. Ltd., Framingham, MA, USA) was used to simultaneously detect the concentration of 25-hydroxyvitamin D_3_ (25(OH)D3), 25-hydroxyvitamin D_2_ (25(OH)D2), and 3-epi-25 hydroxyvitamin D (3-Epi-25(OH)D3). The total 25(OH)D concentration of all samples was quantified by referring to the standard curves generated by serial dilutions of accredited standards (MilliporeSigma, St. Louis, MO, USA). The LC-MS/MS method adopted in this study has been certified by achieving satisfactory performance (within ± 25% of the target value) in the proficiency test provided by Vitamin D External Quality Assessment Scheme (DEQAS, Endocrine Laboratory, Charing Cross Hospital, London, UK) [25]. Furthermore, our previous study has showed that the inter-assay coefficients of variability (*N* = 24) using this method was 9.09% [26].

### Dietary intake assessment for infants and toddlers

Feeding pattern including the duration and degree of breastfeeding exclusivity was recorded for infants aged 6 months or below using the Infant Feeding Category Assessment Tool (FeedCat Tool) designed by University of Ottawa [27]. The FeedCat Tool has been demonstrated by previous studies to have good validity and reliability [27].

Since solid food was mainly introduced after the infants reached 6 months of age, a 24-hour dietary call was adopted to record the dietary intake of infants and toddlers aged 7 to 23 months. Their mother or guardian was interviewed by the trained research staff to report the dietary intake of their child in the past 24 h from the date of the interview. A printed booklet of food portion guide was provided to each parent/guardian to facilitate the portion estimation. Dietary data obtained was then analysed by a nutrition analysis software – Food Processor Nutrition Analysis and Fitness Software version 7.9 (Esha Research, Salem, USA) which takes reference to the USDA food composition table as well as the traditional Chinese and local Hong Kong food composition tables. Using the data from the 24-hour dietary recall, total vitamin D intake was then computed to examine whether the participants had reached the recommended level of daily vitamin D intake. In this study, total vitamin D intake of less than 400 international units (IU) per day was regarded as insufficient total vitamin D intake.

### Other factors that influence vitamin D status

Parents/guardians of the participating infants and toddlers were also asked to report whether they provided any fish liver oil or any form of vitamin supplementation to their child. Data of sun exposure related factors such as outdoor activity pattern and use of sunscreen was collected by a validated questionnaire adopted from the Diet and nutrition survey of infants and young children 2011 from United Kingdom [28]. Maternal demographic including her age, sex, marital status, and parity, and infant’s information including the age and sex were obtained by a questionnaire completed by the mothers. Anthropometric measures such as body weight and body height were also obtained during their visit at the Maternal and Child Health Centre. Body mass index (BMI) was computed by dividing weight in kilograms (kg) by the square of height in meters (m²).

### Data analysis

Descriptive statistics were computed to summarize the demographic characteristics of the mother-child dyads in the study sample. Data were presented as mean (standard deviation (SD)) for continuous variables, median (interquartile range) for skewed variables and frequency (percentage) for categorical variables. Characteristics of infants and toddlers of different vitamin D status were compared using Fisher’s exact test or Chi-square test for categorical variables and Student’s t-test or Wilcoxon ranked-sum tests for continuous variables, whenever it is appropriate.

Associations between vitamin D status and related factors were analysed using regression models after controlling for age and gender of the child. To be specific, multiple linear regressions were used for total serum 25(OH)D concentrations being the dependent variable, and logistic regression was used for vitamin D status being categorical variable. Further analyses were then conducted to examine whether the effects of related factors differ on the concentrations of 25(OH)D3, 25(OH)D2 and 3-Epi-25(OH)D3 of the participants. All statistical tests were 2-sided and a *p*-value < 0.05 was considered statistically significant. Statistical Package for Social Sciences software for Windows (version 26.0, SPSS Inc., Chicago, IL, USA) was used for the statistical analysis.

### Ethics approval

The study was approved by the Hong Kong Department of Health Ethics Committee (Ref no: LM 524/2018) and the Institutional Review Board of the University of Hong Kong/Hospital Authority Hong Kong West Cluster Research Ethics Committee (Ref no.: UW 13–055 and UW 19–497). The research protocol, including the consent procedures, genetic information assessment and vitamin D measurement method, was in accordance with the Declaration of Helsinki. Written informed consent was obtained from all parents/guardians of infants and toddlers, and pregnant women by the research staff.

## Results

**Fig. 1:**
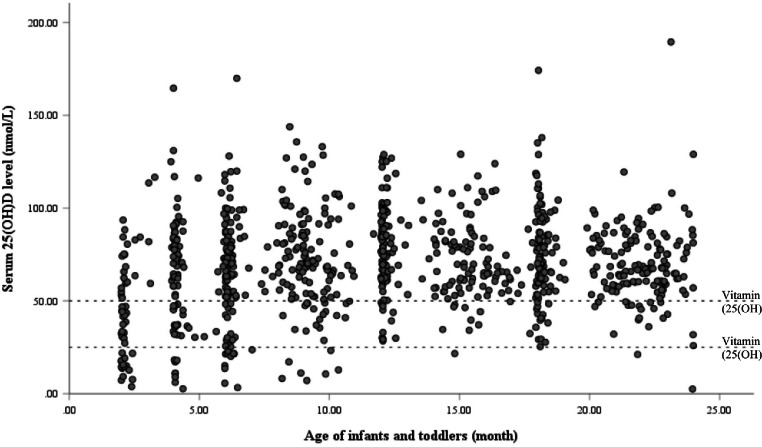
Serum 25(OH)D concentration of enrolled infants and toddlers by their age

A total of 408 infants and 479 toddlers were recruited from different regions of Hong Kong. Among the 887 infants and toddlers recruited, 719 (81.1%) participants were found to have sufficient vitamin D, 124 (14.0%) were vitamin D insufficient and 44 (5.0%) were vitamin D deficient. Figure 1 displays the distribution of serum 25(OH)D concentration of enrolled infants and toddlers by their age. The mean serum 25(OH)D concentration was 62.1nmol/L in younger infants (2 to 6 months), 71.6nmol/L in older infants (7 to 11 months), and 73.2nmol/L in toddlers (12 to 23 months).

The characteristics of the enrolled infants and toddlers are listed in Table 1. The numbers of male and female participants were 474 and 413 respectively, with a ratio of 1.15 to 1. The mean z-score for height and weight were − 0.05 and − 0.007, respectively. About half of the participants came from a family of monthly income higher than HKD$39,999 (USD5128.2) and about 95% of the participant’s mother were married. About one third of the participants had one sibling and more than half did not have any sibling at the time of interview. Preliminary comparative analyses showed that vitamin D deficient and insufficient subjects were significantly younger (*p* < 0.001). Also, the vitamin D status of the participants was significantly associated with their sex (*p* = 0.031) and number of siblings (*p* = 0.003). The proportion of vitamin D insufficient infants and toddlers were higher in girls and those who had siblings.

**Table 1:** Characteristics of participants

		Vitamin D status			
Overall	Total (*N* = 887)	Sufficient (≥ 50nmol/L) (*N* = 719)	Insufficient (25 ≤ 25(OH)D < 50nmol/L) (*N* = 124)	Deficient: (< 25nmol/L) (*N* = 44)	*p*
Age of the child, month, mean (SD)	12.0 (6.3)	12.6 (6.1)	10.6 (6.7)	6.0 (4.8)	< 0.001
Sex, N (%)					0.031
Boys	474 (53.4%)	395 (54.9%)	53 (42.7%)	26 (59.1%)	
Girls	413 (46.6%)	324 (45.1%)	71 (57.3%)	18 (40.9%)	
Maternal age, year, mean (SD)	33.40 (4.34)	33.45 (4.36)	33.60 (3.88)	31.95 (4.90)	0.076
Height, z-score, mean (SD)	-0.05 (1.06)	-0.06 (1.06)	-0.03 (1.12)	-0.09 (0.83)	0.945
Weight, z -score, mean (SD)	-0.007 (0.93)	-0.01 (0.93)	0.02 (0.89)	-0.03 (1.03)	0.938
Birth weight, kg, mean (SD)	3.15 (0.36)	3.14 (0.36)	3.17 (0.37)	3.13 (0.34)	0.725
$Family Income, N (%)					0.576
Low	298 (33.6%)	247 (34.4%)	40 (32.3%)	11 (25.0%)	
Median	142 (16.0%)	109 (15.2%)	24 (19.4%)	9 (20.5%)	
High	419 (47.2%)	342 (47.6%)	57 (46.0%)	20 (45.5%)	
Maternal marital status, N (%)					0.599
Married	842 (94.9%)	682 (94.9%)	120 (96.8%)	40 (90.9%)	
Single/Divorced	39 (4.4%)	32 (4.5%)	4 (3.2%)	3 (6.8%)	
Number of siblings, N (%)					0.003
None	507 (57.2%)	433 (60.2%)	56 (45.2%)	18 (40.9%)	
One	313 (35.3%)	233 (32.4%)	58 (46.8%)	22 (50.0%)	
Two or more	58 (6.5%)	45 (6.3%)	10 (8.1%)	3 (6.8%)	
Month of blood taking, N (%)					0.100
December to February	201 (22.7%)	163 (22.7%)	27 (21.8%)	11 (25.0%)	
March to May	168 (18.9%)	145 (20.2%)	15 (12.1%)	8 (18.2%)	
June to August	203 (22.9%)	152 (21.1%)	41 (33.1%)	10 (22.7%)	
September to November	315 (35.5%)	259 (36.0%)	41 (33.1%)	15 (34.1%)	

Note SD: standard deviation. 39,999; High = > $39,999, Missing information: family income (*n* = 28), maternal marital status (*n* = 6), number of siblings (*n* = 9)

Table 2 displays the vitamin D-related behaviours and dietary pattern of the participants. In average, participants reported to have 2.5 days of outdoor activities in the preceding week. Less than 10% of them have sun cream usage and only 13.5% had consumed vitamin D-containing supplement. For younger infants aged 2 to 6 months, about one third of them had breast milk as their primary food source (32.0%), 30.8% were mixed-fed and 30.0% were formula-fed. For those aged 7 to 23 months, 25.2% were still breastfed and 74.3% were still formula-fed in the past 7 days. The mean total daily vitamin D for the participants was found to be 238.3IU, which is lower than the recommended intake level of 400IU per day. Preliminary comparative analyses suggested that both supplementation practice and dietary pattern can influence the vitamin D status of the participants.

**Table 2:** Vitamin D-related behaviours and dietary pattern of participants (*N* = 887)

	Vitamin D status				
	Total	Sufficient	Insufficient	Deficient	*p*
**Child vitamin D-related behaviours**					
Number of days with outdoor activity in previous week, mean (SD)	2.5 (2.5)	2.6 (2.5)	2.4 (2.5)	2.2 (2.5)	0.459
Sun cream usage in previous week, N (%)					0.215
Yes	63 (7.1%)	56 (7.8%)	6 (4.8%)	1 (2.3%)	
No	814 (91.8%)	654 (91.0%)	118 (95.2%)	42 (95.5%)	
Vitamin D-containing supplementation, N (%)					0.007
Yes	120 (13.5%)	108 (15.0%)	12 (9.7%)	0 (0.0%)	
No	759 (85.6%)	604 (84.0%)	112 (90.3%)	43 (97.7%)	
**Child dietary pattern**					
*2 to 6 months (N = 253)*					
Primary food source, N (%)					< 0.001
Breast milk	81 (32.0%)	23 (13.5%)	29 (58.0%)	29 (90.6%)	
Mixed	78 (30.8%)	64 (37.4%)	13 (26.0%)	1 (3.1%)	
Formula milk	76 (30.0%)	71 (41.5%)	5 (10.0%)	0 (0.0%)	
*7 to 23 months (N = 634)*					
Breastfed in the past 7 days, N (%)					< 0.001
Yes	160 (25.2%)	98 (17.9%)	52 (70.3%)	10 (83.3%)	
No	464 (73.2%)	442 (80.7%)	21 (28.4%)	1 (8.3%)	
Formula-fed in the past 7 days, N (%)					< 0.001
Yes	471 (74.3%)	451 (82.3%)	19 (25.7%)	1 (8.3%)	
No	153 (24.1%)	89 (16.2%)	54 (73.0%)	10 (83.3%)	
Total daily vitamin D intake, IU, mean (SD)	238.3 (226.1)	258.6 (227.1)	112.5 (172.8)	93.1 (168.7)	< 0.001

Note SD: standard deviation, Missing information: sun ream usage in previous week (*n* = 10), vitamin D-containing supplementation (*n* = 8), primary food source (*n* = 18), breastfed in the past 7 days (*n* = 10), formula-fed in the past 7 days (*n* = 10)

Tables 3 and 4 display the biochemical outcomes of the participants and their correlation. The mean total 25(OH)D concentration was 69.7 nmol/L, in which the mean 25(OH)D3, 25(OH)D2, and 3-Epi-25(OH)D3 concentrations were 72.6 nmol/L, 1.8 nmol/L and 4.6 nmol/L, respectively. According to the cut-offs used and recommended in the Consensus Vitamin D position statement from UK National Health Service [24], the prevalence of vitamin D deficiency was 12.6% in younger infants, 5.1% in older infants, and 0.8% in toddlers, and the prevalence of vitamin D insufficiency was 19.8% in younger infants, 12.9% in older infants, and 11.3% in toddlers. The mean albumin, alkaline phosphatase, calcium, and phosphate concentration were 40.8 g/L, 285.8 IU/L, 2.5 mmol/L and 1.8 mmol/L, respectively. Our analyses showed that calcium concentration was significantly correlated with 25(OH)D3, 25(OH)D2, and 3-Epi-25(OH)D3 (*p* < 0.05) but not with the total 25(OH)D concentration. Phosphate concentration was also found to be significantly correlated with 25(OH)D2, and 3-Epi-25(OH)D3 (*p* < 0.01).

**Table 3:** Biochemical outcomes of participants (*N* = 887)

			Vitamin D status			
		Total	Sufficient	Insufficient	Deficient	*p*
	N		Mean (SD)			
Total 25(OH)D, nmol/L	887	69.7 (25.8)	78.4 (19.7)	39.4 (7.4)	14.4 (6.3)	< 0.001
25(OH)D3, nmol/L	887	72.6 (26.2)	81.2 (20.1)	42.0 (9.2)	18.6 (15.3)	< 0.001
25(OH)D2, nmol/L	887	1.8 (3.6)	2.0 (3.8)	1.2 (2.4)	0.5 (1.0)	0.004
3-Epi-25(OH)D3, nmol/L	887	4.6 (4.3)	4.8 (4.4)	3.8 (3.6)	2.6 (3.6)	0.001
Albumin, g/L	876	40.8 (2.4)	40.9 (2.3)	40.7 (2.7)	40.4 (2.9)	0.332
Alkaline phosphatase, IU/L	876	285.8 (94.0)	285.9 (97.5)	277.6 (72.9)	307.2 (86.6)	0.201
Calcium, mmol/L	876	2.5 (0.1)	2.5 (0.1)	2.5 (0.1)	2.5 (0.1)	0.510
Phosphate, mmol/L	876	1.8 (0.2)	1.8 (0.2)	1.9 (0.2)	1.9 (0.2)	0.136

Note SD: standard deviation

**Table 4:** Correlation between biochemical outcomes of participants (*N* = 887)

		*N*	1	2	3	4	5	6	7	8
1	Total 25(OH)D	887	-	0.97***	0.07*	0.02	0.06	-0.06	0.05	0.01
2	25(OH)D3	887		-	-0.06	0.18***	0.05	-0.06	0.07*	0.04
3	25(OH)D2	887			-	0.04	0.08*	-0.04	-0.09*	-0.10**
4	3-Epi-25(OH)D3	887				-	0.04	-0.04	0.07*	0.14***
5	Albumin	876					-	0.01	0.35***	-0.05
6	Alkaline phosphatase	876						-	0.04	-0.03
7	Calcium	876							-	0.35***
8	Phosphate	876								-

Note *: *p* < 0.05; **: *p* < 0.001; ***: *p* < 0.001

Table 5 shows the results of the logistic regression analyses on factors affecting vitamin D insufficient risk. After adjusted for age and gender, lack of supplementation practice (aOR = 2.16), and having siblings (aOR = 2.06) were the major risk factors for vitamin D insufficiency (all *p* < 0.05). Dietary pattern was also a significant risk factor. For participants aged 2 to 6 months, being exclusively breastfed could significantly increase vitamin D insufficiency risk (aOR = 24.91, *p* < 0.001). For those aged 7 to 23 months, our analyses showed that an increment of 100IU in total daily vitamin D intake could significantly lower the vitamin D insufficiency risk (aOR = 0.50, *p* < 0.001). In addition, being breastfed in the past 7 days was also a risk factor (aOR = 7.36, *p* < 0.001).

**Table 5:** Factors affecting vitamin D insufficient risk [Serum 25(OH)D concentration < 50nmol/L] (*N* = 887)

		Crude association		Adjusted model^^^	
	N	OR (95% CI)	*p*	aOR (95% CI)	*p*
Age	887	0.92 (0.89,0.94)	< 0.001	0.91 (0.89,0.94)	< 0.001
Sex	887				
Boys	474	1	-	1	-
Girls	413	1.37 (0.98,1.92)	0.065	1.43 (1.01,2.01)	0.043
Number of days with outdoor activity (as per increment of 1 day)	879	0.96 (0.90,1.03)	0.260	1.01 (0.94,1.08)	0.851
Vitamin D-containing supplementation	879				
Yes	120	1	-	1	-
No	759	2.31 (1.24,4.30)	0.008	2.16 (1.15,4.05)	0.017
Primary food source (2 to 6 months)	235				
Breast milk	81	21.18 (9.38, 47.82)	< 0.001	24.91 (10.27, 60.42)	< 0.001
Mixed	78	1.84 (0.77, 4.40)	0.173	1.63 (0.65, 4.10)	0.3
Formula milk	76	1	-	1	-
Total vitamin D intake less than 400IU per day (7 to 23 months)	634				
Yes	547	3.57 (1.27, 10.03)	0.016	3.60 (1.28, 10.12)	0.015
No	87	1	-	1	-
An increment of 100IU in total daily vitamin D intake (7 to 23 months)	634	0.50 (0.41, 0.62)	< 0.001	0.50 (0.41, 0.62)	< 0.001
Breastfed in the past 7 days (7 to 23 months)	624				
Yes	160	5.04 (2.78, 9.12)	< 0.001	7.36 (3.57, 15.18)	< 0.001
No	464	1	-	1	-
Had any siblings	878				
Yes	371	1.96 (1.39,2.75)	< 0.001	2.06 (1.45,2.93)	< 0.001
No	507	1	-	1	-
Family income status	859				
High income	419	1.09 (0.74,1.61)	0.663	1.05 (0.71,1.57)	0.794
Middle income	142	1.47 (0.90,2.40)	0.128	1.47 (0.88,2.43)	0.138
Low income	298	1	-	1	-
Month of blood taking	887				
December to February	201	1	-	1	-
March to May	168	0.68 (0.39,1.20)	0.181	0.75 (0.42,1.33)	0.322
June to August	203	1.44 (0.90,2.31)	0.133	1.50 (0.92,2.44)	0.103
September to November	315	0.93 (0.59,1.46)	0.746	0.99 (0.62,1.57)	0.954

Note ^^^Adjusted for age and gender

To further delineate the risk factors on the vitamin D status of the participants, a series of linear regression analyses were conducted to examine effects of various factors on the concentrations of total 25(OH)D, 25(OH)D3, 25(OH)D2 and 3-Epi-25(OH)D3. As shown in Table 6, age- and gender-adjusted regression models found that lack of supplementation practice would be significantly negatively associated with the concentrations of total 25(OH)D, 25(OH)D3, 25(OH)D2 and 3-Epi-25(OH)D3 (*p* < 0.05). Factors including being breastfed, having insufficient vitamin D intake, having siblings, family income and month of blood taking affected the concentrations of some vitamin D metabolite. For example, insufficient vitamin D intake only affected the total 25(OH)D (β=-0.09, *p* = 0.020) and 25(OH)D3 concentrations (β=-0.11, *p* = 0.040) but not that of 25(OH)D2 (β = 0.001, *p* = 0.989) and 3-Epi-25(OH)D3 (β=-0.05, *p* = 0.233) among child aged 7 to 23 months.

**Table 6:** Factors affecting total 25(OH)D, 25(OH)D3, 25(OH)D2 and 3-Epi-25(OH)D3 concentrations (*N* = 887)

		Total 25(OH)D		25(OH)D3		25(OH)D2		3-Epi-25(OH)D3	
	*N*	β (95% CI)	*p*	β (95% CI)	*p*	β (95% CI)	*p*	β (95% CI)	*p*
Age	887	0.14 (0.07, 0.20)	< 0.001	0.09 (0.02, 0.16)	0.007	0.22 (0.16, 0.29)	< 0.001	-0.13 (-0.19, -0.06)	< 0.001
Sex	887								
Boys	474	-	-	-	-	-	-	-	-
Girls	413	-0.09 (-0.16, -0.03)	0.005	-0.09 (-0.15, -0.02)	0.008	0.002 (-0.06, 0.07)	0.962	0.05 (-0.02, 0.11)	0.138
Number of days with outdoor activity (as per increment of 1 day)	879	0.03 (-0.04, 0.10)	0.391	0.04 (-0.03, 0.10)	0.274	-0.07 (-0.14, -0.004)	0.037	-0.04 (-0.11, 0.03)	0.230
Vitamin D-containing supplementation	879								
Yes	120	-	-	-	-	-	-	-	-
No	759	-0.12 (-0.19, -0.06)	< 0.001	-0.12 (-0.18, -0.05)	< 0.001	-0.11 (-0.17, -0.05)	< 0.001	-0.10 (-0.17, -0.04)	0.002
Primary food source (2 to 6 months)	235								
Breast milk	81	-0.58 (-0.72, -0.43)	< 0.001	-0.60 (-0.74, -0.46)	< 0.001	0.02 (-0.16, 0.19)	0.862	-0.30 (-0.47, -0.14)	< 0.001
Mixed	78	-0.18 (-0.35, -0.01)	0.040	-0.17 (-0.35, 0.00)	0.047	0.06 (-0.11, 0.23)	0.489	0.03 (-0.14, 0.20)	0.707
Formula milk	76	-	-	-	-	-	-	-	-
Total vitamin D intake less than 400IU per day (7 to 23 months)	634								
Yes	547	-0.09 (-0.17, -0.01)	0.020	-0.11 (-0.19, -0.04)	0.004	0.001 (-0.08, 0.08)	0.989	-0.05 (-0.13, 0.03)	0.233
No	87	-	-	-	-	-	-	-	-
An increment of 100IU in total daily vitamin D intake (7 to 23 months)	634	0.21 (0.13, 0.28)	< 0.001	0.23 (0.16, 0.30)	< 0.001	-0.01 (-0.09, 0.07)	0.786	0.09 (0.02, 0.17)	0.017
Breastfed in the past 7 days (7 to 23 months)	624								
Yes	160	-0.24 (-0.32, -0.15)	< 0.001	-0.23 (-0.31, -0.15)	< 0.001	-0.04 (-0.13, 0.04)	0.320	-0.04 (-0.12, 0.05)	0.404
No	464	-	-	-	-	-	-	-	-
Had any siblings	878								
Yes	371	-0.15 (-0.21, -0.08)	< 0.001	-0.15 (-0.21, -0.08)	< 0.001	0.07 (0.01, 0.14)	0.028	0.02 (-0.05, 0.08)	0.580
No	507	-	-	-	-	-	-	-	-
Family income status	859								
High income	419	-0.07 (-0.14, -0.0003)	0.049	-0.08 (-0.16, -0.01)	0.023	-0.07 (-0.14, 0.01)	0.073	-0.13 (-0.21, -0.06)	< 0.001
Middle income	142	-0.08 (-0.17, 0.02)	0.103	-0.08 (-0.18, 0.01)	0.085	-0.02 (-0.11, 0.08)	0.727	-0.12 (-0.21, -0.03)	0.013
Low income	298	-	-	-	-	-	-	-	-
Month of blood taking	887								
December to February	201	-	-	-	-	-	-	-	-
March to May	168	-0.06 (-0.16, 0.05)	0.284	-0.06 (-0.16, 0.04)	0.238	-0.12 (-0.22, -0.01)	0.025	-0.15 (-0.25, -0.05)	0.003
June to August	203	-0.27 (-0.36, -0.18)	< 0.001	-0.22 (-0.32, -0.13)	< 0.001	-0.04 (-0.14, 0.06)	0.409	0.25 (0.16, 0.35)	< 0.001
September to November	315	-0.18 (-0.27, -0.10)	< 0.001	-0.15 (-0.23, -0.06)	< 0.001	0.02 (-0.06, 0.11)	0.575	0.20 (0.12, 0.29)	< 0.001

Note All β were adjusted for age and gender of the participants

## Discussion

The present study is the first population-representative survey in Hong Kong that examined the vitamin D status among infants and toddlers in Hong Kong, which are known to be one of the most vulnerable groups towards vitamin D deficiency. A comprehensive assessment was conducted on the contributing factors affecting the vitamin D insufficiency risk at different levels. In addition, our study analysed and compared whether 25(OH)D2, 25(OH)D3 and 3-epi25(OH)D3 are likely to be differently influenced by the identified vitamin D risk factors.

In this study, the average 25(OH)D concentration is 62.1 nmol/L in younger infants (2 to 6 months), 71.6 nmol/L in older infants (7 to 11 months), and 73.2 nmol/L in toddlers (12 to 23 months), respectively. The average 25(OH)D concentration in our study is slightly higher than local data conducted in infants with a mean age of 3 months in 2018 (median = 58 nmol/L) [29]. Only a few studies have been carried out globally focusing specifically on the vitamin D status of infants and toddlers. A previous study conducted in Boston, United States found that 12.1% of infants and toddlers (aged 8 to 24 months) had vitamin D concentration of less than 50 nmol/L [14]. The Diet and Nutrition Survey of Infants and Young Children of the UK in 2011 found that 6% of infants (5 to 12 months) and 2% of toddlers (12 to 18 months) were vitamin D deficient (< 25 nmol/L) [30]. According to the multi-centre Chinese survey in 2020, 4.66% of infants (1 to 12 months) and 2.11% of toddlers (13 to 36 months) were found to have vitamin D concentration of less than 30 nmol/L [31]. These suggest that problem of vitamin D deficiency and insufficiency in Hong Kong is still severe, especially among younger infants. It is still crucial to identify key risk factors affecting the vitamin D deficiency risk among these vulnerable groups to facilitate the establishment of guidelines and recommendation for achieving optimal vitamin D concentration among infants and toddlers in Hong Kong.

In line with previous studies, our study found that breastfeeding is a primary risk factor for low vitamin D concentration in infants and toddlers. Although breastfeeding has been suggested to be the most preferred and nutritious food source for infants with its potential health benefits [32, 33], human breast milk is intrinsically limited by a relatively low vitamin D content of approximately 10 to 80 IU/L (about three times less vitamin D than the maternal circulating concentration) [16, 34]. Exclusively breastfed infants are therefore often unable to meet their daily vitamin D requirement without supplementation or adequate sunlight exposure [35, 36]. In view of this, guidelines and recommendations have been made by various governing bodies and society worldwide on vitamin D supplementation for breastfed infants. A fairly large consensus exists that all infants should receive at least 400 IU (10 µg) daily during their first year of life [37–39], or even up to two years old [40, 41]. However, our study indicates that only 13.5% have taken vitamin D-containing supplement at the time of interview. This could be resulted from parental non-compliance, or limited guidelines provided by local practitioners, putting breastfed infants at a higher risk of vitamin D deficiency [42, 43]. Furthermore, it was found in our study that over 85% of the infants and toddlers have insufficient total vitamin D intake (total vitamin D intake less than 400IU per day) [37]. Our analyses found that not only insufficient intake D was a significant risk factor for vitamin D insufficiency, a decrement of 100 IU in total vitamin D intake per day can already increase the vitamin D insufficiency risk significantly. Our findings indicate a necessity for guidelines with detailed recommendation on vitamin D supplementation and dietary intake for infants and toddlers in Hong Kong.

Our analyses also identified the effects of various unmodified factors on vitamin D status in infants and toddlers, including age, gender, and number of siblings. Specifically, our result showed that younger infants (2 to 6 months) have the highest prevalence in vitamin D deficiency in Hong Kong (12.6%). This finding is in line with an age-specific variation of vitamin D status in children reported by a previous Chinese study [44]. It was reported that the prevalence of vitamin D deficiency decreased with age from birth to 6 months and remained with minor fluctuation from 6 to 12 months, followed by continuously decrease after reaching 12 months of age [44]. Consistent with previous studies [45, 46], this study also found gender differences in vitamin D deficiency risk in infants and toddlers. Our results showed that girls were more susceptible to vitamin D deficiency and insufficiency. Possible explanations may be gene-by-sex interaction that potentially affects infants’ susceptibility to vitamin D deficiency [47]. However, as there is no clear evidence over the potentiality of gene-by-sex interaction, there might be other unmeasured factors causing the increased vitamin D insufficiency risk among girls. Our study also found that infants and toddlers who have siblings display a higher risk in vitamin D insufficiency. Such phenomenon could be caused by higher caregiving burden and lower health awareness of the multi-parous mothers [48, 49]. A large-scale family-based cohort study in Korea even observed a familial clustering in vitamin D deficiency [50]. While many studies have indicated that increased outdoor activities can help decrease the risk of vitamin D deficiency in older children and adolescents [51, 52], our findings suggest that might not be same among infants and toddlers. This potentially could relate to the current guidelines that recommend younger children are covered with clothes and avoid direct ultraviolet radiation [41, 53]. Furthermore, although seasonality of vitamin D status has been observed in infants and children in Mainland China and Taiwan [31, 54], we did not observe any seasonal effects on the vitamin D insufficiency risk among infants and toddlers in this study. Both the studies in Mainland China and Taiwan found that infants and children were more susceptible to vitamin D deficiency or insufficiency during spring and winter [31, 54]. The seasonal effect in Hong Kong on vitamin D status may have been offset by other factors such as sun cream usage, outdoor activity patterns, and supplementation practice, and also seasonal variation of sunlight exposure in Hong Kong might not be the same as other parts of world.

Our study revealed that certain overlapping factors, such as age, number of siblings, and vitamin D-containing supplementation, are related to both 25(OH)D2 and 25(OH)D3 in infants and toddlers. However, it also indicated that distinct risk factors may be separately associated with 25(OH)D3 and 25(OH)D2 concentrations in different ways. In our study, the infant’s feeding pattern was associated only with 25(OH)D3, while outdoor activity time was negatively associated only with 25(OH)D2. So far, there is limited study simultaneously discussing the potential factors related to 25(OH)D2 and 25(OH)D3 in children. Only three studies were retrieved, including the Avon longitudinal study in southwest England (*n* = 4,393, mean age: 9 years old) [55], a large Chinese population study about vitamin D status from 30 provinces (*n* = 1,528,685, 0–119 years old) [18], and a recent study about vitamin D2 deficiency in hospitalized children in Hebei, China (*n* = 11,506, 0–15 years old) [56]. The Avon longitudinal study found some common factors that 25(OH)D3 and 25(OH)D2 were both negatively associated with age, female gender, Tanner stage, BMI or waist and maternal education; but displayed inverse associations with vigorous physical activity, equalised household income and winter season [55]. Besides this, this study showed factors related to 25(OH)D3 only (positively associated with vitamin D intake, UVB protection score, outdoor time during summer; negatively associated with non-white ethnicity) and others with 25(OH)D2 only (positively associated with carbohydrate intake; negatively associated with protein intake and parent’s social class, and housing tenure) [55]. The large Chinese population study compared the distribution of a total 25(OH)D and 25(OH)D2 and examined the age, gender and seasonal effects. Both total 25(OH)D and 25(OH)D2 varied with age and sex. The 25(OH)D concentrations were consistently higher in males than in females at all ages, while 25(OH)D2 between the two genders displayed fluctuations among age groups [18]. No significant seasonal variation of 25(OH)D2 was observed in that study [18]. The Hebei local study reported the degree of vitamin D2 deficiency was related to age, but there was no gender difference [56]. The existing data, which is inconsistent and varies significantly, could be attributed to the diverse age groups, ethnicities and other cofounders that have not been investigated. Hence, more comprehensive research is warranted to investigate the vitamin D2 status and more modifiable factors associated with 25(OH)D2 concentrations among different age groups, such as dietary sources or nutrient supplements. In addition, the phenomenon of low vitamin D2 status is common in children with both skeletal and non-skeletal diseases [56]. Different from 25(OH)D3, associations of 25(OH)D2 with many health outcomes in children were in mixed directions [22, 57, 58]. It is necessary to take into account potential confounders in studies on any form of 25(OH)D when investigating the associations between vitamin D status and various research outcomes, which may strengthen the reliability of causality of the associations between vitamin D and specific diseases to provide better guidance for clinical practice.

Our results show the variation of 3-epi-25(OH)D3 is not associated with gender but declines with age and can be modified by the breastfeeding and vitamin D supplement. Thus, oral vitamin D supplements is likely to be exogenous source of 3-epi-25(OH)D3 in infants and toddlers. In our participants, epi-3-25(OH)D3 roughly accounts for 5.8% (mean: 4.6 nmol/L) of the total detected vitamin D concentration. Previous work showed higher average concentration of 3-epi-25(OH)D3 was 11.4 nmol/L in infants ≤ 1 year of age and 5.4 nmol/L in toddlers 1–2 years of age [59]. 3-epi-25(OH)D3 binds vitamin D binding protein at 36–46% and vitamin D receptor at 2–3% as compared with non-epimer 25(OH)D3 [15]. To accurately interpret the real functional vitamin D during early childhood, it is necessary to exclude the interferences of epi-3-25(OH)D3.

This study is a multi-centre population study with a relatively representative sample size, reporting the vitamin D status in infants and toddlers in the local setting. This study also focused on early childhood vitamin D status, presenting insights on strategies for preventing infants and toddlers from vitamin D deficiency. Besides this, factors impacting the vitamin D metabolites, 25(OH)D3, 25(OH)D2 and 3-epi-25(OH)D3, were separately considered to better understand the factors associated with the total vitamin D status. The standardised LC/MS-MS measurement method allowed us to accurately quantify 25(OH)D3 and avoided the misclassification of vitamin D status. There are also several limitations to be considered. Firstly, this was a cross-sectional study and therefore we were not able to establish a causal relationship between different risk factors and vitamin D status. Secondly, the selection bias may be generated as the study participants were only recruited from the Maternal and Child Health Centre (MCHC). Those who visited private health sectors for health services were not covered in the sampling of this study. They may be different in health awareness, dietary patterns and supplementation practice. However, over 90% of the infants and toddlers were reported to visit MCHCs in Hong Kong. Therefore, the sample recruited in this study can be considered representative of the target populations in Hong Kong. Thirdly, we did not measure the maternal vitamin D concentration, which is a known factor to affect the vitamin D status of infants as maternal vitamin D status determines the neonatal vitamin D status and breastmilk vitamin D [60]. A more comprehensive study with longitudinal assessments could further strengthen the evidence on the contributing factors to vitamin D status in infants and toddlers in Hong Kong.

Our study indicated that mode of feeding, dietary intake, and use of vitamin D-containing nutritional supplement are key factors influencing the vitamin D concentration of infants and toddlers. To address this issue, it is crucial to provide additional guidelines and support to ensure these groups meet their daily vitamin D intake requirements. In addition to guideline provision, policies such as universal supplementation and government-subsidized supplement purchases can be implemented to combat vitamin D deficiency and prevent rickets among infants and toddlers in Hong Kong. Further studies are needed to confirm the evidence on factors affecting vitamin D status in infants and toddlers.
