# Correction: Vitamin D status and its determinants among Chinese infants and toddlers in Hong Kong

**DOI:** 10.1007/s00394-025-03811-w

**Published:** 2025-10-28

**Authors:** Keith T. S. Tung, Hung Kwan So, Chen Chen, Joanna Y. L. Tung, Hing Wai Tsang, Rosa S. Wong, Sophie S. F. Leung, Calvin K. M. Cheung, Albert Lee, Jason C. S. Yam, Wing Cheong Leung, Patrick Ip

**Affiliations:** 1 Department of Paediatrics and Adolescent Medicine, School of Clinical Medicine, The University of Hong Kong, Hong Kong SAR, China; 2 Department of Paediatrics, Hong Kong Children’s Hospital, Hospital Authority, Hong Kong SAR, China; 3 Department of Special Education & Counselling, The Education University of Hong Kong, Hong Kong SAR, China; 4 Department of Paediatrics, Faculty of Medicine, The Chinese University of Hong Kong, Hong Kong SAR, China; 5 Jockey Club School of Public Health and Primary Care, The Chinese University of Hong Kong, Hong Kong SAR, China; 6 Department of Ophthalmology and Visual Sciences, The Chinese University of Hong Kong, Hong Kong SAR, China; 7 Department of Obstetrics and Gynaecology, Kwong Wah Hospital, Hong Kong SAR, China

**Correction to: European Journal of Nutrition (2025) 64:220** 10.1007/s00394-025-03691-0

In the original version of this article, author did not acknowledge their previous study using part of the dataset. Last paragraph in the introduction section, which previously read as

Our study has 3 objectives (1) to report the prevalence of vitamin D deficiency and insufficiency in infants and toddlers in Hong Kong; (2) to determine the risk factors associated with vitamin D deficiency and insufficiency; (3) to investigate whether the concentrations of 25(OH)D2, 25(OH)D3 and 3-epi25(OH)D3 are influenced by the identified vitamin D risk factors.

but should have read

A pilot study was conducted by our team in 2021 involving 208 young infants (aged 2 to 6 months) to perform a cumulative risk assessment on their vitamin D insufficiency risk [1]. This study made use of data from participants in that pilot study as well as the remaining recruited participants to examine the below objectives. Our study has 3 objectives (1) to report the prevalence of vitamin D deficiency and insufficiency in infants and toddlers in Hong Kong; (2) to determine the risk factors associated with vitamin D deficiency and insufficiency; (3) to investigate whether the concentrations of 25(OH)D2, 25(OH)D3 and 3-epi25(OH)D3 are influenced by the identified vitamin D risk factors.

The Funding information section was missing and should have read

This work was supported by Health and Medical Research Fund (HMRF), the Health Bureau, Hong Kong SAR Government (Reference No.: Vit D-HKU).
